# Catalytic activities of a highly efficient cocaine hydrolase for hydrolysis of biologically active cocaine metabolites norcocaine and benzoylecgonine

**DOI:** 10.1038/s41598-022-27280-x

**Published:** 2023-01-12

**Authors:** Linyue Shang, Zhenyu Jin, Huimei Wei, Shawn Park, Chang-Guo Zhan, Fang Zheng

**Affiliations:** 1 Molecular Modeling and Biopharmaceutical Center, College of Pharmacy, University of Kentucky, 789 South Limestone Street, Lexington, KY 40536 USA; 2 Department of Pharmaceutical Sciences, College of Pharmacy, University of Kentucky, 789 South Limestone Street, Lexington, KY 40536 USA

**Keywords:** Biochemistry, Drug discovery

## Abstract

Cocaine is a widely abused, hepatotoxic drug without an FDA-approved pharmacotherapy specific for cocaine addiction or overdose. It is recognized as a promising therapeutic strategy to accelerate cocaine metabolism which can convert cocaine to pharmacologically inactive metabolite(s) using an efficient cocaine-metabolizing enzyme. Our previous studies have successfully designed and discovered a highly efficient cocaine hydrolase, denoted as CocH5-Fc(M6), capable of rapidly hydrolyzing cocaine at the benzoyl ester moiety. In the present study, we determined the kinetic parameters of CocH5-Fc(M6) against norcocaine (*k*_cat_ = 9,210 min^−1^, *K*_M_ = 20.9 μM, and *k*_cat_/*K*_M_ = 1.87 × 10^5^ min^−1^ M^−1^) and benzoylecgonine (*k*_cat_ = 158 min^−1^, *K*_M_ = 286 μM, and *k*_cat_/*K*_M_ = 5.5 × 10^5^ min^−1^ M^−1^) for the first time. Further in vivo studies have demonstrated that CocH5-Fc(M6) can effectively accelerate clearance of not only cocaine, but also norcocaine (known as a cocaine metabolite which is more toxic than cocaine itself) and benzoylecgonine (known as an unfavorable long-lasting metabolite with some long-term toxicity concerns) in rats. Due to the desired high catalytic activity against norcocaine, CocH5-Fc(M6) is capable of quickly detoxifying both cocaine and its more toxic metabolite norcocaine after intraperitoneally administering lethal dose of 60 or 180 mg/kg cocaine. In addition, the ability of CocH5-Fc(M6) to accelerate clearance of benzoylecgonine should also be valuable for the use of CocH5-Fc(M6) in treatment of cocaine use disorder.

## Introduction

Cocaine is a hepatotoxic drug^1^ abused widely. Despite of extensive efforts in development of therapeutics for treatment of cocaine use disorders, there is still no U.S. Food and Drug Administration (FDA)-approved pharmacotherapy specific for cocaine addiction or overdose. Traditional pharmacodynamic approaches that aim to antagonize the action of cocaine have been very elusive^2–5^. It is highly desired to develop novel therapeutic strategies. Recently, accelerating cocaine metabolism using an efficient cocaine-metabolizing enzyme has been recognized as a promising strategy^6–11^.

**Figure 1:**
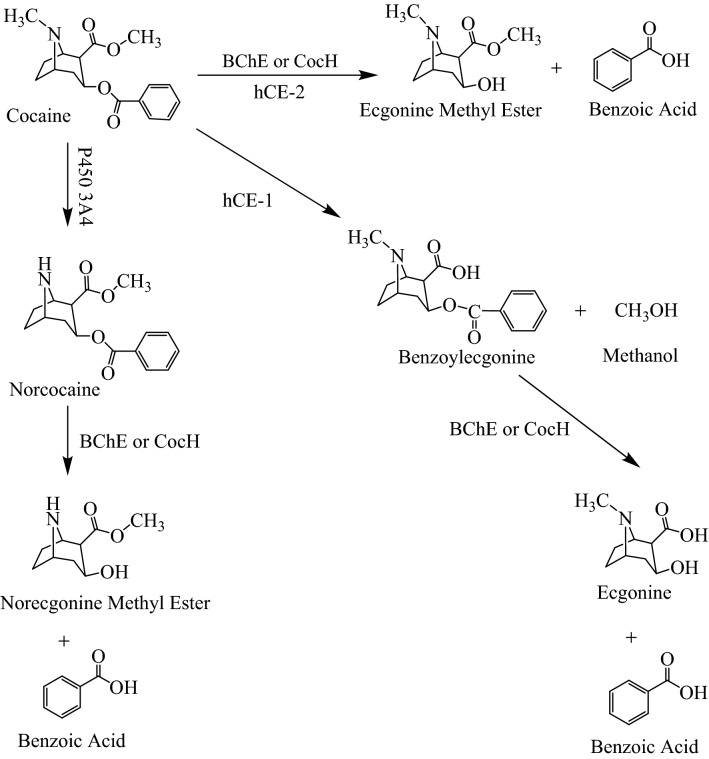
Metabolic pathways of cocaine in human. The metabolic enzymes include the endogenous enzymes butyrylcholinesterase (BChE), liver carboxylesterase-1 (hCE-1), liver carboxylesterase-2 (hCE-2), and cytochrome (CYP) P450 3A4, as well as exogenous enzyme cocaine hydrolase (CocH) if a CocH is used in clinic in the future.

Cocaine is metabolized via three pathways shown in Fig. 1. First, cocaine is hydrolyzed by butyrylcholinesterase (BChE) at the benzoyl ester group to produce metabolite ecgonine methyl ester (EME) which is known as a pharmacologically inactive metabolite. In addition to BChE, human liver carboxylesterase-2 (hCE-2) also contributes to this metabolic pathway.

Another metabolic pathway is cocaine methyl ester hydrolysis catalyzed by human liver carboxylesterase-1 (hCE-1), producing benzoylecgonine^12^. Benzoylecgonine was reported to affect cerebral artery^13^ and be a potent vasoconstrictor in literatures^14,15^, but was considered inactive in some other publications^16,17^. Although it is disputable whether benzoylecgonine is mainly responsible for the long-term toxicity of cocaine^14^, benzoylecgonine lasts in human body for a much longer time than cocaine itself^1,2^. For this reason, it is a common clinical practice to use benzoylecgonine as a cocaine use marker in the pre-employment drug test.

In addition, cocaine is also oxidized by liver microsomal cytochrome P450 (3A4), and this metabolic pathway produces metabolite norcocaine which has similar physiological effects to that of cocaine^18,19^. Notably, norcocaine is more toxic than cocaine itself, with LD_50_ = 49.71 mg/kg for norcocaine (when administered intraperitoneally or IP) in mice compared to LD_50_ = 93.0 mg/kg for cocaine (IP) in mice^20^. Based on the relative magnitudes of the LD_50_ values for cocaine and norcocaine in the same strain of mice, norcocaine is nearly two-fold more toxic than cocaine. The known cocaine hepatotoxicity is attributed to the further oxidation of norcocaine^21^.

Based on the properties of the cocaine metabolites, EME is the least pharmacologically active metabolite of cocaine, and it may even cause vasodilation^22^. It would be ideal to develop a therapeutic enzyme (with a similar function like the endogenous BChE) which can accelerate hydrolysis of cocaine at the benzoyl ester moiety. However, the catalytic activity of wild-type BChE against (-)-cocaine (the naturally occurring cocaine) (*k*_cat_ = 4.1 min^−1^ and *K*_M_ = 4.5 μM)^23–27^ is too low to be useful as a therapeutic enzyme candidate.

For rational design of a human BChE mutant with a considerably improved catalytic activity against (-)-cocaine, our group developed a unique computational strategy known as the structure-and-mechanism-based computational design^28–38^. The computational design was followed by wet experiments in vitro and in vivo, leading to discovery of various BChE mutants with a considerably improved catalytic efficiency against (-)-cocaine^9,28–35,39^. These BChE mutants are also known as cocaine hydrolases (CocHs), as they are highly efficient against cocaine. Within all the CocHs reported so far, the most potent one is CocH5 (which is the A199S/F227A/P285A/S287G/A328W/Y332G mutant of human BChE)^35^. Most recently, a long-acting Fc-fusion protein formulation of CocH5, denoted as CocH5-Fc(M6), has been developed as a clinical candidate^9^. It has been demonstrated that even at a single modest dose of 3 mg/kg, CocH5-Fc(M6) can significantly and effectively accelerate metabolism of cocaine in rats for at least 60 days^9^. Through accelerating cocaine metabolism, CocH5-Fc(M6) was able to completely block the toxicity and physiological effects induced by a lethal dose of cocaine (60 mg/kg, IP) in rats^7,9^. It has also been demonstrated in rats that CocH5-Fc(M6) can effectively protect the dopaminergic system and help the cocaine-altered dopamine transporter (DAT) distribution to recover by preventing the dopaminergic system from further damage by cocaine^7,8,40^.

Further, we wanted to know whether CocH5-Fc(M6) can also effectively accelerate further metabolism of norcocaine (as it is even more toxic than cocaine^20^) and benzoylecgonine (as it might be responsible for the long-term toxicity of cocaine^14^). This is particularly important for treatment of cocaine toxicity (overdose conditions). When a patient with cocaine overdose seeks toxicity treatment, a portion of cocaine has already been metabolized to norcocaine and benzoylecgonine. Hence, a truly effective enzyme therapy for cocaine toxicity should be able to effectively accelerate hydrolysis of not only cocaine itself, but also norcocaine and benzoylecgonine, especially norcocaine. In the present study, we have determined the catalytic activities of CocH5-Fc(M6) against norcocaine and benzoylecgonine in vitro and in vivo, demonstrating that CocH5-Fc(M6) is indeed capable of accelerating hydrolysis of not only cocaine itself, but also norcocaine and benzoylecgonine. Thus, CocH5-Fc(M6) is a potent cocaine antidote.

## Results

### Computational insights

**Figure 2:**
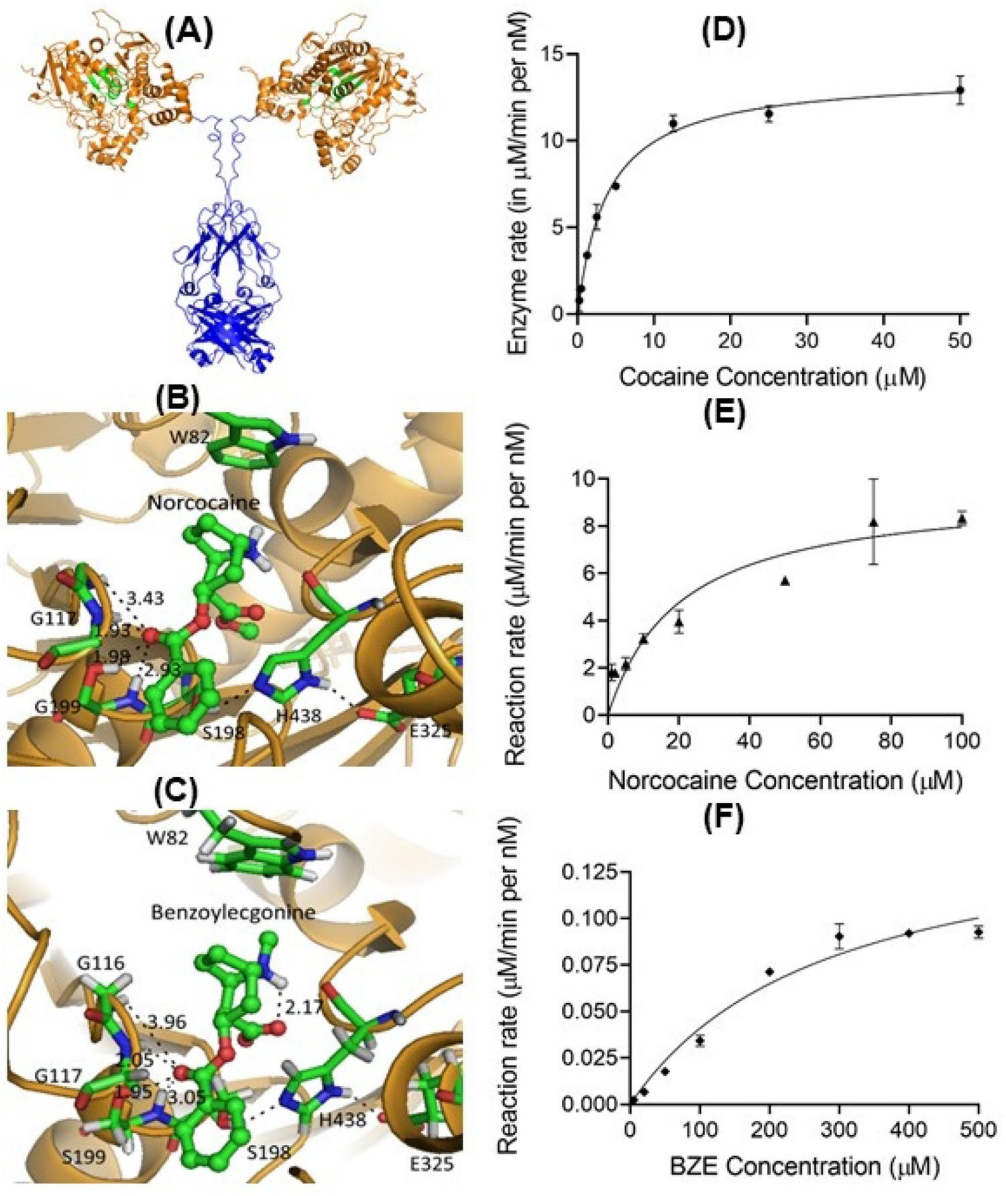
CocH5-substrate (norcocaine or benzoylecgonine) binding and the Michaelis–Menten kinetics. (**A**) The general shape of the CocH5-Fc(M6) fusion protein dimer in which the binding sites of CocH5 (the A199S/F227A/P285A/S287G/A328W/Y332G mutant of human BChE) are highlighted. The CocH5 portion was based on the X-ray crystal structure of BChE (pdb identification code: 1P0P), and the Fc(M6) portion was based on the X-ray crystal structure of Fc (pdb identification code: 4N0U). (**B**) CocH5-norcocaine binding mode. (**C**) CocH5-benzoylecgonine binding mode. (**D**) CocH5-catalyzed hydrolysis of (-)-cocaine (data from previous report^9^). (**E**) CocH5-catalyzed hydrolysis of norcocaine. (**F**) CocH5-catalyzed hydrolysis of benzoylecgonine. All measurements were performed in triplicate.

Molecular docking was performed to examine whether the active site in CocH5 portion of CocH5-Fc(M6) (Fig. 2A) can bind with norcocaine or benzoylecgonine in a binding mode suitable for the desired enzymatic hydrolysis. As seen in Fig. 2, both norcocaine and benzoylecgonine can bind to the active site with a pose suitable for the desired chemical reactions. Particularly, the carbonyl carbon of the benzoyl ester group is reasonably close to the hydroxyl oxygen of S198 (part of the well-known catalytic triad consisting of S198, H438, and E325), and the corresponding carbonyl oxygen atom of the benzoyl ester group stays in the well-known oxyanion hole consisting of G116, G117, and A199 of wild-type BChE (Fig. 2B,C). Previous modeling and enzyme redesign studies^28,31,33–35,37,41^ revealed that, for a given substrate, the hydrogen bonding of the carbonyl oxygen of the substrate with the oxyanion hole of BChE will help to stabilize the transition state and, thus, decrease the energy barrier for the enzymatic hydrolysis. Notably, in CocH5 (the A199S/F227A/P285A/S287G/A328W/Y332G mutant of human BChE), A199 becomes S199, and the A199S mutation produces one more hydrogen bond between the carbonyl oxygen and the hydroxyl group of S199 side chain. This additional hydrogen bond made us to hypothesize that CocH5-Fc(M6) may be more active than wild-type BChE in enzymatic hydrolysis of norcocaine and benzoylecgonine.

### Kinetic parameters

In light of the insights from computational modeling, we carried out in vitro experimental assays to determine the actual catalytic activities of CocH5-Fc(M6) against norcocaine and benzoylecgonine through Michaelis–Menten kinetic analysis. The obtained concentration-dependent reaction rates against norcocaine and benzoylecgonine are shown in Fig. 2E,F, respectively, in comparison with previously determined kinetic data against (-)-cocaine^9^ (Fig. 2D). The obtained kinetic parameters are summarized in Table 1.

**Table 1:** Kinetic parameters of CocH5-Fc(M6) against (-)-cocaine, norcocaine, and benzoylecgonine at 37 °C.

	Cocaine^a^	Norcocaine	Benzoylecgonine
*k*_cat_ (min^−1^)	13,800	9210	158
*K*_M_ (μM)	3.89	20.9	286
*k*_cat_/*K*_M_ (min^−1^ M^−1^)	3.55 × 10^9^	4.41 × 10^8^	5.5 × 10^5^

^a^Kinetic parameters for cocaine came from our previous report^9^.

As seen in Table 1, for CocH5-Fc(M6)-catalyzed hydrolysis of norcocaine, we obtained *k*_cat_ = 9,210 min^−1^ and *K*_M_ = 20.9 μM, giving a catalytic efficiency (*k*_cat_/*K*_M_) of 4.41 × 10^8^ min^−1^ M^−1^. Compared to wild-type BChE-catalyzed hydrolysis of norcocaine (*k*_cat_ = 2.8 min^−1^, *K*_M_ = 15 μM, and *k*_cat_/*K*_M_ = 1.87 × 10^5^ min^−1^ M^−1^)^42^, CocH5-Fc(M6) has a ~ 2,360-fold improved catalytic efficiency against norcocaine.

For CocH5-Fc(M6)-catalyzed hydrolysis of benzoylecgonine, the Michaelis–Menten kinetic analysis revealed that *k*_cat_ = 158 min^−1^, *K*_M_ = 286 μM, and *k*_cat_/*K*_M_ = 5.5 × 10^5^ min^−1^ M^−1^. Compared to the previously determined catalytic activity of wild-type BChE against benzoylecgonine (*k*_cat_ = 3.6 min^−1^, *K*_M_ = 83 μM, and *k*_cat_/*K*_M_ = 4.34 × 10^4^ min^−1^ M^−1^)^43^, CocH5-Fc(M6) has a ~ 13-fold improved catalytic efficiency (*k*_cat_/*K*_M_) against benzoylecgonine.

**Figure 3:**
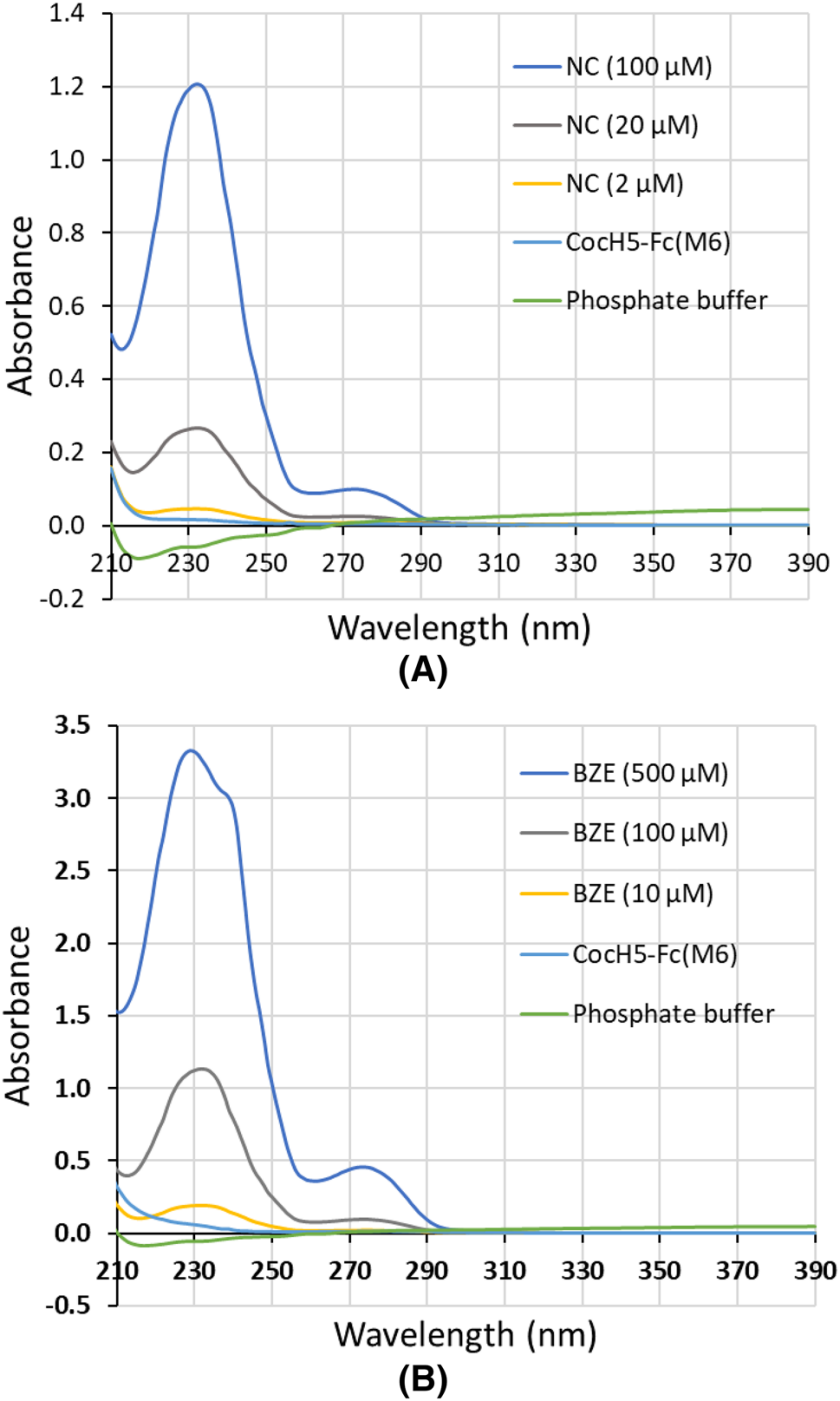
UV–visible absorption spectra of potassium phosphate buffer (blank), CocH5-Fc(M6) and substrates in buffer. (**A**) Norcocaine (NC). (**B**) Benzoylecgonine (BZE).

The above measurement was based on the observation that both norcocaine and benzoylecgonine had ultraviolet (UV) absorbance between 210 and 390 nm with a peak at ~ 230 nm, the absorption at 230 nm is proportional to the concentration of norcocaine/benzoylecgonine, and that the UV absorbance of either phosphate buffer (for the blank) or CocH5-Fc(M6) had no interference with the UV absorbance of norcocaine or benzoylecgonine at 230 nm, as shown in Fig. 3.

### CocH5-Fc(M6)-accelerated clearance of norcocaine and benzoylecgonine in rats

**Figure 4:**
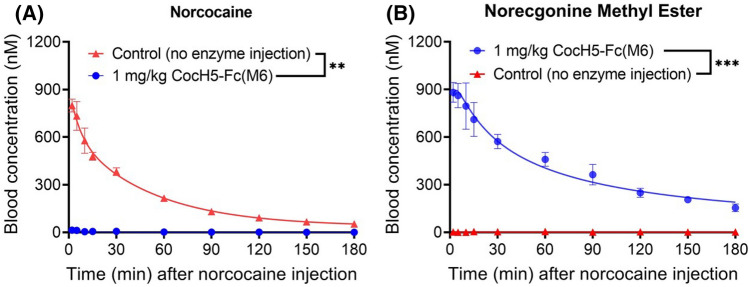
CocH5-Fc(M6)-accelerated clearance of norcocaine in rats in comparison with untreated group (n = 4 per group). Time-dependent concentrations of (**A**) norcocaine and (**B**) norecgonine methyl ester. Statistical significance (paired *t* test using the GraphPad Prism 8.0 software): *p < 0.05; **p < 0.01; ***p < 0.001; ****p < 0.0001.

**Figure 5:**
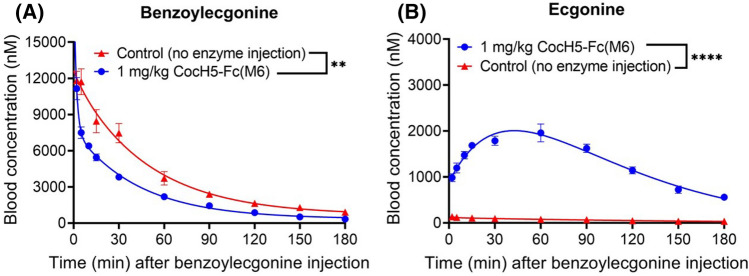
CocH5-Fc(M6)-accelerated clearance of benzoylecgonine in rats (n = 4 per group). Time-dependent concentrations of (**A**) benzoylecgonine and (**B**) ecgonine. The benzoylecgonine and ecgonine data for control (untreated rats) were reported previously^43^. Statistical significance (paired *t* test using the GraphPad Prism 8.0 software): *p < 0.05; **p < 0.01; ***p < 0.001; ****p < 0.0001.

To examine whether CocH5-Fc(M6) can effectively accelerate hydrolysis of norcocaine and benzoylecgonine in vivo, rats (n = 4 per group) were administered intravenously (IV, via tail vein) with 1 mg/kg CocH5-Fc(M6), followed by intravenous (IV) administration of 2 mg/kg norcocaine or 2 mg/kg benzoylecgonine. Blood samples were collected at various time points (2, 5, 10, 15, 30, 60, 90, 120, 150, and 180 min) after the IV administration of norcocaine or benzoylecgonine and analyzed by our previously established LC–MS/MS method^44^. The obtained in vivo data are shown in Figs. 4 and 5.

As shown in Fig. 4A, compared to the pharmacokinetic (PK) profile of norcocaine without CocH5-Fc(M6) administration (control rats), administration of 1 mg/kg CocH5-Fc(M6) remarkably accelerated norcocaine clearance. The average plasma concentration of norcocaine was only ~ 14 nM at the first time point (2 min) compared to the corresponding average plasma norcocaine concentration of ~ 799 nM in the control rats. The average plasma norcocaine concentration in the CocH5-Fc(M6)-treated rats was only ~ 2% of that in the control rats. In other words, ~ 98% of norcocaine was hydrolyzed by CocH5-Fc(M6) within ~ 2 min.

Further, the product of CocH5-Fc(M6)-catalyzed hydrolysis of norcocaine is norecgonine methyl ester shown in Fig. 1. As seen in Fig. 4B, in the control rats (without enzyme injection), the measured plasma concentrations of norecgonine methyl ester were all below the detection limit (~ 10 nM). In the CocH5-Fc(M6)-treated rats, the average plasma concentration of norecgonine methyl ester was as high as ~ 882 nM, which is consistent with the CocH5-Fc(M6)-catalyzed hydrolysis of norcocaine.

According to the data shown in Fig. 5, CocH5-Fc(M6) administration also significantly accelerated benzoylecgonine clearance (Fig. 5A), while significantly increasing the plasma concentrations of ecgonine—the product of the benzoylecgonine hydrolysis (Fig. 5B). Unlike norcocaine, benzoylecgonine has a zwitterion structure that is very stable in the body. Due to the slower clearance rate and relatively lower catalytic efficiency of CocH5-Fc(M6) against benzoylecgonine, the plasma ecgonine concentration increased gradually with a peak at 30 ~ 60 min, in contrast to the case of norcocaine that the metabolite norecgonine methyl ester peaked immediately after the enzyme administration. Eventually, the plasma concentration of the metabolite ecgonine or norecgonine methyl ester decreased over time when its clearance rate became greater than the corresponding generation rate in the body.

### Effects of CocH5-Fc(M6) on the metabolic profile of a lethal dose of cocaine (60 or 180 mg/kg, IP) in rats

**Figure 6:**
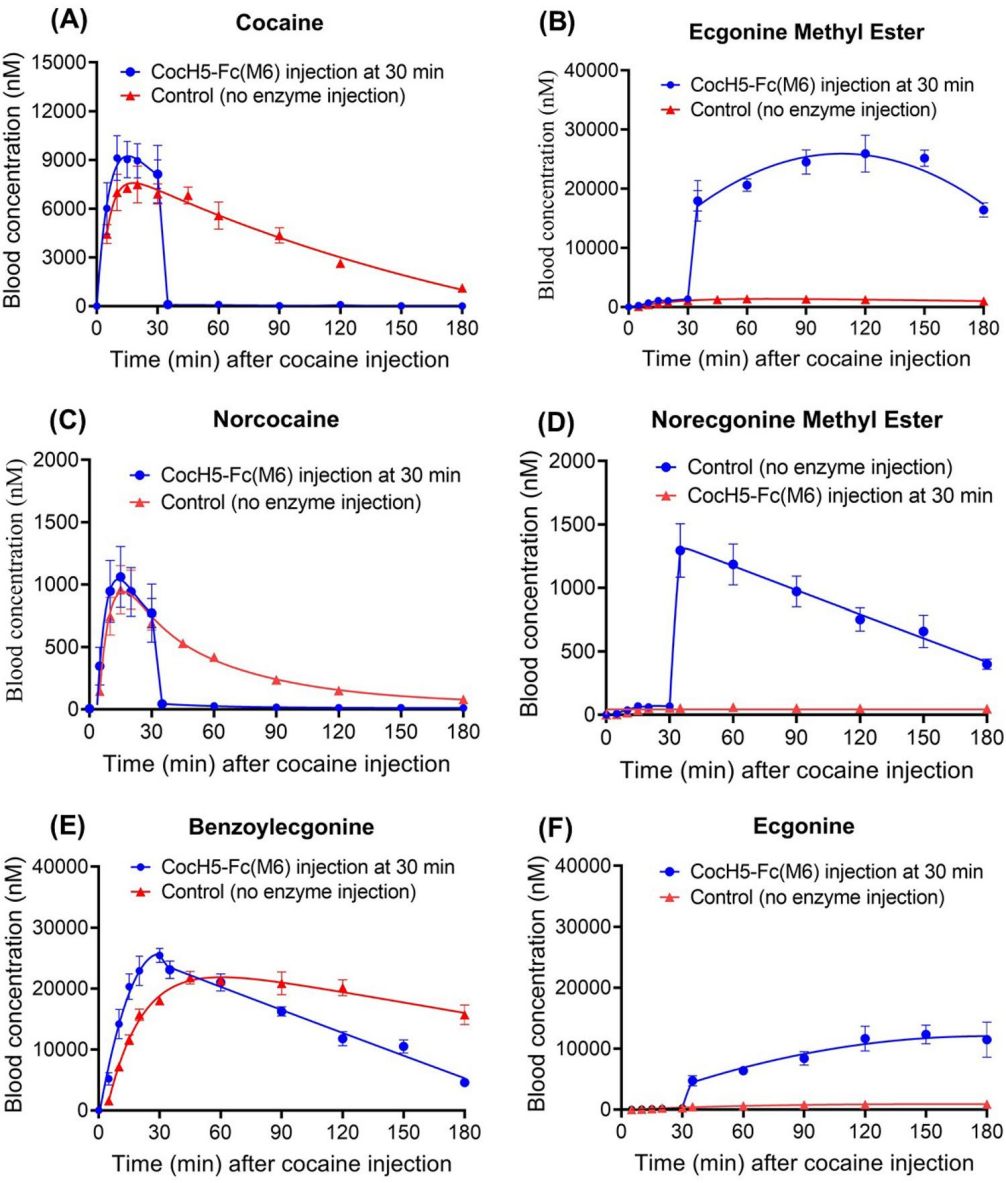
Time-dependent concentrations of (**A**) (-)-cocaine, (**B**) ecgonine methyl ester (EME), (**C**) norcocaine, (**D**) norecgonine methyl ester, (**E**) benzoylecgonine, and (**F**) ecgonine. 1 mg/kg CocH5-Fc(M6) was administered (IV) at 30 min after IP administration of 60 mg/kg cocaine for the treatment rats (n = 4). The corresponding data for the control rats were reported previously^45^.

Cocaine is metabolized to norcocaine via oxidation by cytochrome P450 3A4 which is primarily found in liver. This shows that norcocaine concentration in plasma has been observed much higher in rats received intraperitoneal (IP) injection of cocaine compared to those received intravenous (IV) injection of cocaine. Our previous studies^10,45^ revealed that intraperitoneal (IP) administration of 60 mg/kg cocaine was lethal, most of the rats (75%) had convulsion and 25% rats eventually died after convulsion^46^. According to the previously determined metabolic profile of cocaine in the survived rats (in the untreated group), IP administration of 60 mg/kg cocaine resulted in high concentrations of norcocaine in plasma, with a peak concentration at ~ 20 min after the cocaine administration^45^. So, we wanted to know whether CocH5-Fc(M6) can effectively accelerate clearance of cocaine-converted norcocaine in comparison with the previously determined metabolic profile of 60 mg/kg cocaine (IP) in the untreated rats. For this purpose, we tested a post-treatment model, with a group of rats administered 60 mg/kg cocaine (IP), followed by IV administration of 1 mg/kg CocH5-Fc(M6) at 30 min after the cocaine administration. Blood samples were collected at various time points (5, 10, 15, 20, 30, 35, 60, 90, 120, 150, and 180 min) after the cocaine administration (Particularly for the 30 min time point, blood samples for the LC–MS/MS analysis^44^ were collected right before the enzyme injection). Depicted in Fig. 6 is the obtained metabolic profile of 60 mg/kg cocaine (IP) in the CocH5-Fc(M6)-treated rats (n = 4) in comparison with previously determined metabolic profile of 60 mg/kg cocaine (IP) in the untreated rats.

As seen in Fig. 6A, immediately after the enzyme administration, the plasma cocaine concentration sharply dropped to the detection limit at the first time point (5 min after the enzyme administration or 35 min after the cocaine administration). Meanwhile, the plasma concentration of EME (product of the CocH5-Fc(M6)-catalyzed cocaine hydrolysis) sharply increased to a very high level (~ 18 μM; see Fig. 6B).

As mentioned above, plasma concentration of norcocaine rapidly increased after the cocaine administration (Fig. 6C). Immediately after the CocH5-Fc(M6) administration, the plasma concentration of norcocaine sharply dropped to the detection limit at the first time point (5 min after the enzyme administration or 35 min after the cocaine administration). Meanwhile, the plasma concentration of norecgonine methyl ester (the product of CocH5-Fc(M6)-catalyzed norcocaine hydrolysis) sharply increased to a very high level (~ 1.3 μM; see Fig. 6D).

In addition, according to Fig. 6E, after the cocaine administration, the plasma concentration of benzoylecgonine gradually increased to a peak at ~ 45 min and then slowly decreased in the untreated rats due to its metabolic stability in the body. In the CocH5-Fc(M6)-treated rats, the plasma concentration of benzoylecgonine decreased faster at the first time point (5 min after the enzyme administration or 35 min after the cocaine administration), suggesting the CocH5-Fc(M6)-catalyzed benzoylecgonine hydrolysis. The CocH5-Fc(M6)-catalyzed benzoylecgonine hydrolysis is consistent with the observation (Fig. 6F) that the plasma concentrations of ecgonine (the product of CocH5-Fc(M6)-catalyzed benzoylecgonine hydrolysis) largely increased at all time points (30–180 min) after the enzyme administration at 30 min.

**Figure 7:**
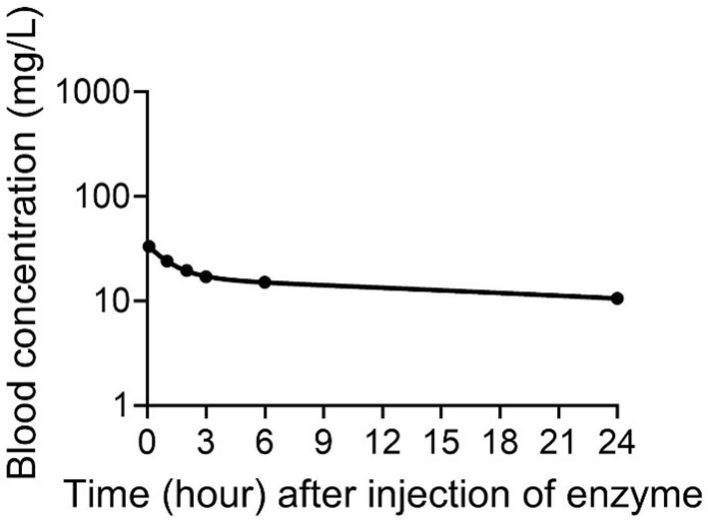
Serum concentration of CocH5-Fc(M6) after IV administration of 1 mg/kg CocH5-Fc(M6) in rats (n = 4).

As shown in our previous report^9^, CocH5-Fc(M6) has a blood elimination half-life of 229 h in rats. We also measured the active CocH5-Fc(M6) concentrations in serum samples collected from these four CocH5-Fc(M6)-treated rats at various time points after the CocH5-Fc(M6) administration in this study. As shown in Fig. 7, the average plasma concentration of CocH5-Fc(M6) was ~ 33.3 mg/L at the first time point (5 min) after the enzyme administration and slowly decreased to ~ 17.0 mg/L at 3 h (or 180 min) and ~ 11 mg/L at 24 h (or 1440 min). So, for all the data shown in Figs. 4, 5, 6 in the CocH5-Fc(M6)-treated rats, the average plasma concentration of CocH5-Fc(M6) was between ~ 33.3 mg/L and ~ 17.0 mg/L, which further confirms that the accelerated clearance of cocaine, norcocaine, and benzoylecgonine was indeed due to the presence of CocH5-Fc(M6) in the blood.

**Figure 8:**
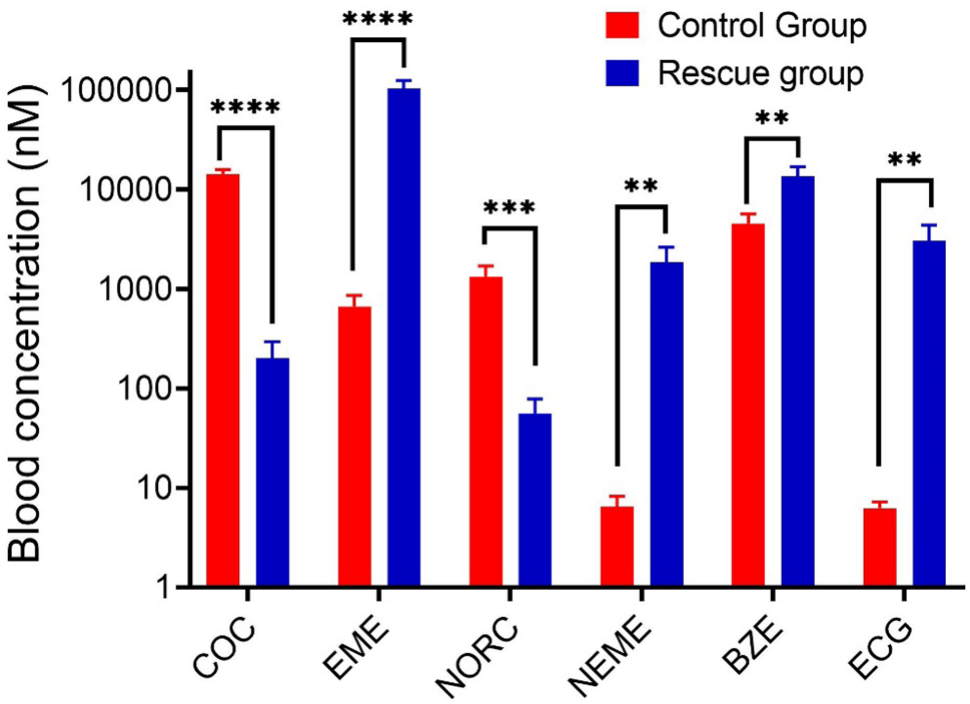
Blood concentrations of cocaine (COC) and its metabolites (*EME* ecgonine methyl ester; *NOC* norcocaine; *NEME* norecgonine methyl ester; *BZE* benzoylecgonine; *ECG* ecgonine) after IP administration of 180 mg/kg cocaine in the control rats and CocH5-Fc(M6)-rescued rats (n = 4 per group) with enzyme administration after the onset of cocaine-induced convulsion (*i.e.* 2–4 min after 180 mg/kg cocaine, IP). For the control rats, blood samples were collected at the time (~ 4 min) right after death (the red bars). For the CocH5-Fc(M6)-rescued rats, blood samples were collected at 10 min after IV administration of 1 mg/kg CocH5-Fc(M6). Statistical analysis (*t* test using the GraphPad Prism 8.0 software): *p < 0.05; **p < 0.01; ***p < 0.001; ****p < 0.0001.

Finally, we tested the effect of CocH5-Fc(M6) on cocaine toxicity in rats by intraperitoneal administration of 180 mg/kg cocaine (LD_100_). The intraperitoneal LD_50_ and LD_100_ of cocaine in Sprague–Dawley rats are 73 and 100 mg/kg, respectively^45^. According to our previous results, all rats showed convulsion at 2.78 ± 1.03 min and died at 4.07 ± 1.87 min after being injected 180 mg/kg cocaine (IP)^46,47^. In the present investigation, we determined the blood concentrations of cocaine and its metabolites in the rats (n = 4 as control group) given 180 mg/kg cocaine (IP) right after their death (Fig. 8). For the group of rats (n = 4) administered 180 mg/kg cocaine (IP) followed by 1 mg/kg CocH5-Fc(M6) (IV) for rescue after the onset of cocaine-induced convulsion (*i.e*. 2–4 min after the cocaine injection), we examined the blood concentrations of cocaine and its metabolites at 10 min after the enzyme administration. The selected time point corresponded to the peak time (10 ~ 15 min) for the blood concentrations of cocaine and its metabolites after the cocaine injection. The obtained blood concentrations of cocaine (0.20 μM) and norcocaine (0.056 μM) in the enzyme-treated rats were only 1.4% and 3.9%, respectively, of the corresponding blood concentrations (14.3 and 1.3 μM for cocaine and norcocaine, respectively) in the control group of rats. Plasma benzoylecgonine concentration (7.3 μM) in the rescue group of rats (at 10 min after the enzyme administration or 12–14 min after the cocaine administration) was slightly higher than that (4.6 μM) in the control group of rats (at ~ 4 min, the average rat death time, after the cocaine administration), which is not surprising due to a couple of reasons. First, plasma benzoylecgonine concentration at 12–14 min after cocaine administration would be expected to be roughly one order of magnitude higher than that at ~ 4 min after cocaine administration, if CocH5-Fc(M6) did not affect plasma benzoylecgonine concentration at all, in light of the data shown in Fig. 6. In fact, CocH5-Fc(M6) converted > 90% cocaine to EME (Fig. 8). In addition, the catalytic efficiency of CocH5-Fc(M6) against benzoylecgonine is not high enough to hydrolyze benzoylecgonine more rapidly than the benzoylecgonine generation in the body within the first 10 min after the enzyme administration. However, the remarkably higher metabolite concentrations of ecgonine methyl ester (103.3 *vs* 0.67 μM), norecgonine methyl ester (1.86 *vs* 0.007 μM), and ecgonine (3.07 *vs* 0.006 μM) in CocH5-Fc(M6) treated rats demonstrated that the enzyme indeed effectively hydrolyzed cocaine, norcocaine, and benzoylecgonine. Notably, 103.3 μM ecgonine methyl ester was detected in blood collected from the enzyme-treated rats, indicating that CocH5-Fc(M6) powerfully eliminated cocaine by converting more than seven-fold amount of cocaine to ecgonine methyl ester compared to the plasma cocaine concentration (14.3 μM, n = 4) which resulted in the rat death. All CocH5-Fc(M6)-treated rats survived and returned to normal walk at about 1–2 min after IV injection of 1 mg/kg CocH5-Fc(M6).

## Discussion

CocH5-Fc(M6) is currently the most efficient, long-acting cocaine hydrolase catalyzing hydrolysis of cocaine at the benzoyl ester moiety, and its catalytic activity against cocaine has been reported in our previous publication^9^. The catalytic activities of CocH5-Fc(M6) against norcocaine and benzoylecgonine were determined for the first time in this study. Based on the data obtained, CocH5-Fc(M6) can also effectively catalyze the benzoyl ester hydrolysis of norcocaine and benzoylecgonine in vitro with respectively ~ 2360-fold and ~ 13-fold improved catalytic efficiencies compared to wild-type BChE. These findings are significant for the development of an enzyme therapy to treat cocaine overdose. As well-known, cocaine benzoyl ester hydrolysis is the most favorable pathway for cocaine metabolism. Accelerating cocaine hydrolysis at the benzoyl ester moiety will rapidly detoxify cocaine in the body. However, the other two metabolic pathways of cocaine shown in Fig. 1 will also produce unfavorable metabolites norcocaine and benzoylecgonine prior to the enzyme treatment for cocaine overdose. Particularly, norcocaine is nearly two-fold more toxic than cocaine itself, and the toxicity of norcocaine contributes to the overall toxicity of cocaine^20,45^. Hence, an ideal enzyme therapy for cocaine overdose treatment would use an enzyme which can efficiently hydrolyze not only cocaine, but also norcocaine at the benzoyl ester group. With the desired catalytic activity against norcocaine, our in vivo results demonstrated that CocH5-Fc(M6) can be expected to powerfully detoxify not only cocaine itself, but also its toxic metabolite norcocaine. Benzoylecgonine is not toxic, but it is also an unfavorable cocaine metabolite with some long-term toxicity concerns associated with its very long elimination half-life in human body. The ability of CocH5-Fc(M6) to accelerate benzoylecgonine clearance from the circulatory system should also be beneficial for therapeutic treatment of cocaine use disorder using CocH5-Fc(M6).

## Materials and methods

### Materials

The purified CocH5-Fc(M6) protein was prepared according to the protocol in our previous study^9^. Controlled substances, including cocaine, norcocaine, and benzoylecgonine, were all provided by the Drug Supply Program of the National Institute on Drug Abuse (Bethesda, MD).

Male Sprague-Darley rats (with a body weight of 200–270 g) were ordered from Envigo (Indianapolis, IN). All the in vivo experiments were carried out in our animal lab within the animal laboratories of the Division of Laboratory Animal Resources (DLAR) facility (PHS assurance number A3336-01; USDA number 61-R-0002; AAALAC, Intl. Unit # 13) at the University of Kentucky, with the DLAR staff providing veterinary care and animal husbandry services. All rats were housed in clean, adequately-sized, stainless-steel cages at 21–22 °C and were allowed ad libitum access to food and water. All animal experiments were performed in accordance with the Guide for the Care and Use of Laboratory Animals as adopted and promulgated by the National Institutes of Health (NIH), and were in fact also consistent with the ARRIVE (Animal Research: Reporting of In Vivo Experiments) guidelines (https://arriveguidelines.org**)**. The animal procedures used in this study had been approved by the University of Kentucky’s Institutional Animal Care and Use Committee (IACUC).

All other general supplies for the experimental studies were obtained from Sigma-Aldrich (St. Louis, MO) or Thermo Fisher Scientific (Waltham, MA).

### Molecular modeling

The binding modes of norcocaine and benzoylecgonine with CocH5 (the A199S/F227A/P285A/S287G/A328W/Y332G mutant of human BChE) were modeled by using the AutoDock 4.2 program^48^ and the X-ray crystal structure of the protein^49^ deposited in the Protein Data Bank (pdb identification code: 1P0P), as we did for other protein–ligand binding systems. During the molecular docking process, the Solis and Wets local search method^50^ and the Lamarkian genetic algorithm (LGA)^48^ were used for the conformational/pose search in order obtain protein–ligand binding structures with the lowest binding free energies.

### In vitro activity assays

UV–Vis absorbance spectra of norcocaine, benzoylecgonine, potassium phosphate buffer and CocH5-Fc(M6) was recorded by using a SpectraMax M5 (Molecular Devices, San Jose, CA). The catalytic activities of CocH5-Fc(M6) against norcocaine and benzoylecgonine were determined by UV–Vis spectrophotometric assays using a GENios Pro Microplate Reader (TECAN, Research Triangle Park, NC) with the XFluor software, as used in our previous studies on wild-type BChE^42,43^. The CocH5-Fc(M6)-catalyzed hydrolysis of norcocaine or benzoylecgonine was initiated by adding 180 μl of substrate (norcocaine or benzoylecgonine in 0.1 M potassium phosphate buffer at pH 7.4) solution with varying concentration to 20 μl of CocH5-Fc(M6) solution. The reaction temperature was 37 °C. The initial rates of the hydrolysis reaction in various initial substrate concentrations were calculated by following the change in the intrinsic absorbance peak of norcocaine or benzoylecgonine (all at 230 nm)^42,43^. All the in vitro activity assays were carried out in triplicate, and the obtained initial reaction rates were analyzed according to the Michaelis–Menten kinetics that allowed us to determine the kinetic parameters (*V*_max_ and *K*_M_ values). Based on the determined *V*_max_ and enzyme concentration [E], catalytic rate constant *k*_cat_ can be evaluated: *k*_cat_ = *V*_max_/[E].

### In vivo activity assays against norcocaine, benzoylecgonine, and cocaine

Rats (n = 4 per group) were administered with CocH5-Fc(M6) at a dose of 1 mg/kg (IV, via tail vein) 1 min before IV administration of 2 mg/kg norcocaine or 2 mg/kg benzoylecgonine. For one cocaine detoxification experiment (post-treatment), a group of four rats (n = 4) were administered with 60 mg/kg cocaine (IP). These rats were administered (IV) with 1 mg/kg CocH5-Fc(M6) at 30 min after the cocaine administration. For another cocaine detoxification experiment (post-treatment or rescue), a group of four rats (n = 4) were administered 180 mg/kg cocaine (IP); after rats started convulsion, 1 mg/kg CocH5-Fc(M6) was intravenously administered. For each experiment, about 75 μl of blood samples were collected from saphenous veins into capillary tubes and mixed immediately with 100 µl of paraoxon (250 µM paraoxon, 10 U/ml heparin in 0.1% formic acid) at various time points after the drug (norcocaine or benzoylecgonine or cocaine) administration. The collected blood samples were stored at − 80 °C until analysis by using our previously reported LC–MS/MS method^44^ for simultaneously determining the concentrations of cocaine and/or all metabolites in blood samples. Besides, additional blood samples were also collected from the saphenous veins of these experimental rats in order to know the actual CocH5-Fc(M6) concentrations in serum samples. The collected serum samples were analyzed for active CocH5-Fc(M6) concentrations by using a sensitive radiometric assay with [^3^H](-)-cocaine (with ^3^H labeled on its benzene ring) as used in our previous studies^9,31,35^.

### Statistical analysis

Comparison between two different groups (treatment and control) in the time-dependent in vivo data for CocH5-Fc(M6)-catalyzed hydrolysis of cocaine, norcocaine, or benzoylecgonine was carried out by paired *t*-test. The statistical analysis was performed using the GraphPad Prism 8.0 (GraphPad, La Jolla, CA. https://www.graphpad.com/updates/prism-8-release-notes). The accepted level of statistical significance was *p* < 0.05 (*).

## Data Availability

The datasets used and/or analyzed during the current study available from the corresponding authors on reasonable request.
